# Storage effect on olive oil phenols: cultivar-specific responses

**DOI:** 10.3389/fnut.2024.1382551

**Published:** 2024-07-10

**Authors:** Mario Vendrell Calatayud, Xueqi Li, Stefano Brizzolara, Pietro Tonutti, Selina C. Wang

**Affiliations:** ^1^Crop Science Research Center, Scuola Superiore Sant'Anna, Pisa, Italy; ^2^Department of Food Science and Technology, University of California Davis, Davis, CA, United States

**Keywords:** oxidation, olive cultivars, phenols, oil composition, quality

## Abstract

**Introduction:**

Olive oil is a widely recognized and appreciated food commodity, its quality and health benefits can be compromised when the oil goes through oxidative processes that may occur during production and storage. This study aimed to investigate the effects of the olive genotype on polar phenolic content after seven months of storage.

**Methods:**

Oil produced from eight different olive cultivars (Leccino, Leccio del Corno, Moraiolo, Frantoio, Bianchera, Pendolino, Maurino, and Caninese) grown in southern Tuscany, Italy, were subjected to chemical analysis such as free fatty acids, peroxide value, K232 and K268, phenolics and UPLC-DAD at the beginning of the trial (Control) and seven months later (Stored).

**Results and Conclusions:**

Free fatty acids, peroxide values, K232 and K268, significantly increased, suggesting heightened hydrolysis and oxidation after storage. A cultivar effect was observed, with Leccino, Moraiolo, and Pendolino showing less susceptibility to oxidation (low differences between Control and Stored). In contrast, others (Bianchera and Caninese) are more affected (higher differences between Control and Stored). Phenolics analysis supports this observation, revealing that samples with higher resistance to oxidation exhibit elevated levels of hydroxytyrosol, tyrosol, vanillic acid, caffeic acid, p-coumaric acid, and ferulic acid. Principal Component Analysis highlights that Bianchera and Caninese cultivars correlate with rutin, tyrosol, and pinoresinol. As this research delves into the intricate relationship between genotype diversity, phenolic composition, and oxidative stability, a nuanced understanding emerges, shedding light on how different cultivars may present varying compositions and concentrations of phenols, ultimately influencing the oil’s resistance to the oxidation that occurred during storage.

## 1 Introduction

Ensuring food safety and quality is paramount to consumers and public authorities due to its direct impact on consumer health. A comprehensive approach to food safety policies, encompassing every stage of the food chain through traceability, is essential. Factors influencing food safety, including conservation methods and chemical composition, play a crucial role in ensuring the quality and safety of food products (1). The inquiry into the quality of olive oil, as posed by Psomiadou and Tsimidou (2), is open to discussion since it depends on how one interprets what quality means. Due to high production costs and limited supplies, olive oil’s evaluation and classification are of primary importance. Over the last decades, the spectrum of techniques and standards for quality assessments has expanded and improved. According to the Commission of European Communities (3), standards were needed to classify the oil depending on its quality. The evaluation establishes this classification of chemical and purity parameters as the standards to determine olive oil quality (free fatty acids, peroxide value, spectrophotometric absorbance K232 and K268, fatty acids profile, and sensory analysis). These factors significantly impact the oil’s flavor, texture, and color, as well as its natural antioxidants and other minor components contributing to its health benefits and preservation (4). Even though oxidative status is considered an important quality trait, the existing legislation often overlooks the in-depth consideration of it, a critical factor that significantly influences the sensorial and nutritional characteristics and shelf-life characteristics of olive oil (5). Many extra virgin olive oil (EVOO) producers and researchers typically consider 12 to 18 months as the maximum commercial storage duration from bottling to consumption. However, the guidelines set by the International Olive Council (6) specify that the maximum commercial storage duration from bottling to consumption for olive oil is 24 months. The International Olive Council (7) classification for extra virgin olive oil indicates that it should have a maximum Free Fatty Acid (FFA) content of 0.8%, Peroxide Value (PV) below 20 meq O2/kg, K232 below 2.50, and K268 below 0.22. The overall quality of the oil, spanning from fruit production to oil consumption, is intricately linked to its oxidative stability, which is crucial in shaping the evolution of flavor, taste, color, and the content of antioxidants (4).

Quality maintenance of olive oil is influenced by multiple factors, among which oil chemical composition stands out as a primary determinant. Inherent factors such as the composition of fatty acids or the presence of antioxidants play a direct role in determining olive oil’s vulnerability to degradation as it ages. During storage, depending on packaging and environment, olive oils experience compositional changes primarily driven by oxidation processes, particularly the interaction between oxygen and fatty acids (8). Oxidation in olive oil can lead to the formation of different undesirable, low molecular weight compounds, such as volatile aldehydes, hydroperoxides, and carbonyls, which can affect the aroma, flavor, and nutritional value of the oil (8, 9). Another co-agent of processes leading to changes in the olive oil can be its microbiota, primarily consisting of yeasts, which can either enhance the oil by hydrolyzing bitter secoiridoid compounds or harm it by degrading its quality by increasing the oxidative parameters (peroxide value, free fatty acids, K232 and K268) (10, 11).

The rich tapestry of olive cultivars reflects a vast genotype diversity, contributing to the heterogeneity in the chemical composition and characteristics of olive oil (12–14). Numerous studies have highlighted that key factors affecting the qualitative and quantitative variations of phenolic compounds in olive oil include genotype (cultivar), climatic and agronomic conditions, edaphic factors, and the employed extraction technology (13, 15). Prior research has often focused on a limited set of genotypes, encompassing either traditional cultivars with regional significance in olive oil production or newly developed cultivars from breeding programs (16, 17). Consequently, a comprehensive assessment and classification of a diverse range of olive cultivars based on the phenolic profiles of monovarietal oils have yet to be extensively documented.

The considerable variability in phenolic families underscores the noteworthy role of secoiridoids, specifically oleuropein and its hydrolyzed derivates. This group of compounds, found in high concentrations in olive oil, has been extensively studied due to the compelling evidence of its health-promoting properties (18). Phenols emerge as pivotal players not only in shaping the distinctive flavor profiles of olive oils but also in safeguarding their resistance to oxidation and lipid degradation (19, 20). Phenolic compounds exhibit the capability to contribute a hydrogen atom to the lipid radical generated during lipid oxidation (21). Studies show that secoiridoid derivatives play a crucial role in enhancing oil stability by binding to the hydroxyl terminal of the lipid radical more efficiently compared to the rest of the phenols found in olive oil, preventing the lipids from being oxidized (22, 23).

This study had three primary objectives: (a) assess the oxidative state of eight distinct Italian olive cultivars by examining chemical parameters before and after storage; (b) analyze the phenol composition of each cultivar before and after storage; (c) investigate changes in phenolic content and examine how variations in these compounds correlate with higher or lower oxidative stability, particularly across different cultivars.

## 2 Materials and methods

### 2.1 Oil samples and storage

The olives originated from orchards located in southern Tuscany, and the oil was manufactured on a semi-industrial level (processing 1,000 kilograms of olives per hour) at the Frantoio Rossi orchards and mill facilities in Scansano (Grosseto GR, Italy). The olive oil was extracted through mechanical processes. The olives were harvested between September and October 2022, with geographical coordinates of 42.64500511746627 latitude and 11.360858291024662 longitude. Three bottles from three different batches were obtained per each cultivar. The sourced cultivars are Leccino, Leccio del Corno, Moraiolo, Frantoio, Bianchera, Pendolino, Maurino, and Caninese, with a ripening index between 2 and 3. Six 20 mL vials containing 18 mL of olive oil and 2 mL of headspace, were prepared per each cultivar. Three vials were used to perform the analysis at the initial acquisition of the oil (Control), and the other three vials were evaluated after a 7-month storage period (Stored). The oil was stored in complete darkness at a temperature of 20°C.

### 2.2 Chemical quality parameters

Olive oil quality parameters, such as free fatty acids (FFA), peroxide value (PV), K232, and K268, were evaluated according to the official methods described in Regulation EC 2568/91 of the Commission of the European Union (3).

The determination of free fatty acids (FFA) content involves dissolving 8 g of oil in a 20 mL mixture of ethanol-ether (1:1 v/v) that has been previously neutralized. The sample is titrated with potassium hydroxide, and the acidity is expressed as a percentage of oleic acid.

Peroxide value (PV) is measured as milliequivalent of active oxygen per kilogram of oil. For this, 1.2–2.0 g of oil is dissolved in chloroform and acetic acid mixture (2:3 v/v). KI saturated solution and deionized water are added, and the sample is titrated with sodium thiosulphate using starch as an indicator.

The extinction coefficients K232 and K268 are calculated through spectrophotometric examination in the ultraviolet range. In this process, 100 mg of oil is dissolved in 25 mL of isooctane, and the extinction of the solution is determined at specified wavelengths using a quartz cell with a 1 cm optical path in a Genesys 10S UV–VIS spectrophotometer (Thermo Scientific, Waltham, MA, US). Specific extinctions are then calculated from the spectrophotometer readings.

### 2.3 UPLC-DAD analysis of polar phenols

Polar phenolic compounds were extracted from oil samples based on the solid-phase extraction (SPE) method provided by Gutierrez-Rosales et al. (25) with some modifications. The oil sample (0.75 g) and internal standard solution (0.15 mL, 6.75 × 10–2 mg/mL of hydroxyphenyl-acetic acid, 1.35 mg/kg final concentration) were dissolved in hexane (2 mL). A 1,000 mg/6 mL dio-bonded phase cartridge (Thermo Scientific, Waltham, MA, US) was put in a VacElut Manifold (Agilent Technologies, Santa Clara, CA, US) and conditioned with methanol (2 mL) and hexane (2 mL) consecutively. After the oil solution was applied to the diol cartridge, the cartridge was washed with hexane (2 mL) twice and hexane/ethyl acetate (2 mL, 90:10, v/v) once. The cartridge was then eluted with methanol (2.6 mL + 1 mL), and the solvent was evaporated in a rotatory evaporator at room temperature under vacuum until dry. Finally, the residue was reconstituted with methanol/water (0.25 mL, 1:1, v/v) for injection.

After the reconstitution, the sample was injected into a 5 μm, 250 mm × 4.6 mm C18 column (Agilent Technologies, Santa Clara, CA, US). It was used for the analysis of Ultra-Performance Liquid Chromatography (UPLC) (Infinity 1,290, Agilent Technologies, Santa Clara, CA, US). The sample injection was 20 μL, and the flow rate was 1.0 mL/min. In this analysis, the mobile phase A was water/acetic acid (98:2, v/v), and B was methanol/acetonitrile (1:1, v/v). The solvent gradient changed according to the following conditions: from 0 to 25 min, 95% A – 5% B to 70% A − 30% B; from 25 min to 50 min to 65% A – 35% B; from 50 min to 65 min to 30% A – 70% B; from 65 min to 70 min, to 100% B; the gradient was then brought back to 95% A – 5% B in 5 min. The Diode Array Detector (DAD) was set at 280 nm and 340 nm. Phenolic compounds were identified by comparing retention times with standard compounds, and the quantification was determined by using relative concentration to the concentration of IS.

### 2.4 Statistical analysis

Results are reported as mean ± standard deviation (*n* = 3) of chemical data and polar phenols detected. The statistical analysis was carried out using R software (24), version 1.1.463-2009-2018 R-studio, Inc.

The statistical tools used are Principal Component Analysis (PCA), ANOVA, and Tukey range test, which were used in chemical quality parameters and polar phenols data. Differences with *p* < 0.05 were considered significant in the ANOVA analysis. Heatmap representation of the escalated fold change analysis (Equation 1) was used to better visualize differences between time 0 (Control) and time 1 (Stored) in the analyzed chemical parameters.

(1) $$\log_{2}\left(\frac{\mathrm{Treated}\;\mathrm{sample}}{\mathrm{Control}\;\mathrm{sample}}\right)$$

## 3 Results and discussion

### 3.1 Chemical analysis

The chemical parameters (Table 1) of olive oil changed after storage, with a general increase in the evaluated parameters in every analyzed cultivar. The observed increases in free fatty acids, peroxide value, and specific absorbance coefficients at 232 nm (K232) and 268 nm (K268) in the olive oil samples collectively signify an increased rate of oxidation. As stated by Paradiso et al. (26), an increase in the FFA can produce an increase in primary and secondary oxidation products (due to its pro-oxidant activity), which are related to rancid or fatty flavors. The peroxide value and K232 reflect the early stages of oxidation, with higher values suggesting increased formation of unstable peroxides. Additionally, higher K268 points to heightened levels of conjugated trienes, indicative of advanced oxidative processes affecting unsaturated fatty acids and, consequently, producing off-flavors such as rancid aromas (25, 27, 28). According to the International Olive Council (29), the initial assessment of some of the parameters at Control aligns with the parameters for extra virgin olive oil (EVOO). Specifically, the peroxide value (PV) is within the EVOO range, and the free fatty acids (FFA) and K232 values are very close to meeting these classification standards. The K268 value, however, corresponds to that of refined olive oil. The evaluation of the Control concluded that the olive oil was not EVOO. After storage, the oil exhibits a significant decline in quality. FFA levels fall within the classification for ordinary virgin oil (≤ 3.3%), and the peroxide value is higher than 20 meq O2/kg, meeting the lampante olive oil criteria, while both K232 and K268 values are no longer confirmed to the EVOO category.

**Table 1:** Chemical evaluation of control and stored oil samples.

Cultivars	Treatment*	FFA	PV	K232	K268
Bianchera	Control	0.844 ± 0.045^b^	13.648 ± 1.970^b^	2.758 ± 0.307^b^	0.788 ± 0.032^b^
	Stored	3.235 ± 0.197^a^	31.782 ± 2.804^a^	6.003 ± 0.352^a^	2.253 ± 0.208^a^
Caninese	Control	0.828 ± 0.026^b^	10.723 ± 1.646^b^	2.977 ± 0.355^b^	0.978 ± 0.019^b^
	Stored	3.052 ± 0.222^a^	26.688 ± 2.248^a^	5.883 ± 0.107^a^	3.274 ± 0.123^a^
Frantoio	Control	0.565 ± 0.046^b^	10.932 ± 1.677^b^	2.302 ± 0.253^b^	0.635 ± 0.017^b^
	Stored	1.772 ± 0.191^a^	23.868 ± 2.588^a^	3.628 ± 0.145^a^	1.912 ± 0.010^a^
Leccino	Control	0.551 ± 0.024^b^	16.883 ± 2.590^b^	2.767 ± 0.224^b^	0.755 ± 0.016^b^
	Stored	1.219 ± 0.162^a^	28.337 ± 3.303^a^	3.773 ± 0.142^a^	1.552 ± 0.260^a^
Leccio del corno	Control	1.107 ± 0.168^b^	10.892 ± 1.671^b^	2.506 ± 0.283^b^	0.771 ± 0.018^b^
	Stored	2.674 ± 0.318^a^	25.727 ± 3.764^a^	4.203 ± 0.352^a^	2.028 ± 0.197^a^
Maurino	Control	0.569 ± 0.025^b^	14.834 ± 2.276^b^	3.187 ± 0.289^b^	0.694 ± 0.012^b^
	Stored	1.631 ± 0.368^a^	30.745 ± 3.643^a^	4.849 ± 0.231^a^	1.896 ± 0.182^a^
Moraiolo	Control	1.120 ± 0.073^b^	10.033 ± 1.540^b^	2.962 ± 0.254^b^	0.754 ± 0.016^b^
	Stored	2.773 ± 0.215^a^	17.054 ± 2.599^a^	4.081 ± 0.244^a^	1.647 ± 0.282^a^
Pendolino	Control	0.557 ± 0.029^b^	12.849 ± 2.093^b^	2.659 ± 0.122^b^	0.531 ± 0.029^b^
	Stored	1.963 ± 0.379^a^	22.851 ± 3.967^a^	3.413 ± 0.208^a^	1.572 ± 0.028^a^

Spectrophotometric indexes (absorbance at 232 and 268 nm), PV (Peroxides) (*meq* O_2_/100g), and FFA (Free Fatty Acids) (mg (KOH)/g) of the oil samples are reported. Results are expressed as mean ± SD (*n* = 3). Different letters indicate statistically significant differences (*p* < 0.05). Treatment*: Control refers to oil analyzed soon after the extraction, and ‘Stored’ refers to oil analyzed after the seventh month of storage.

**Figure 1:**
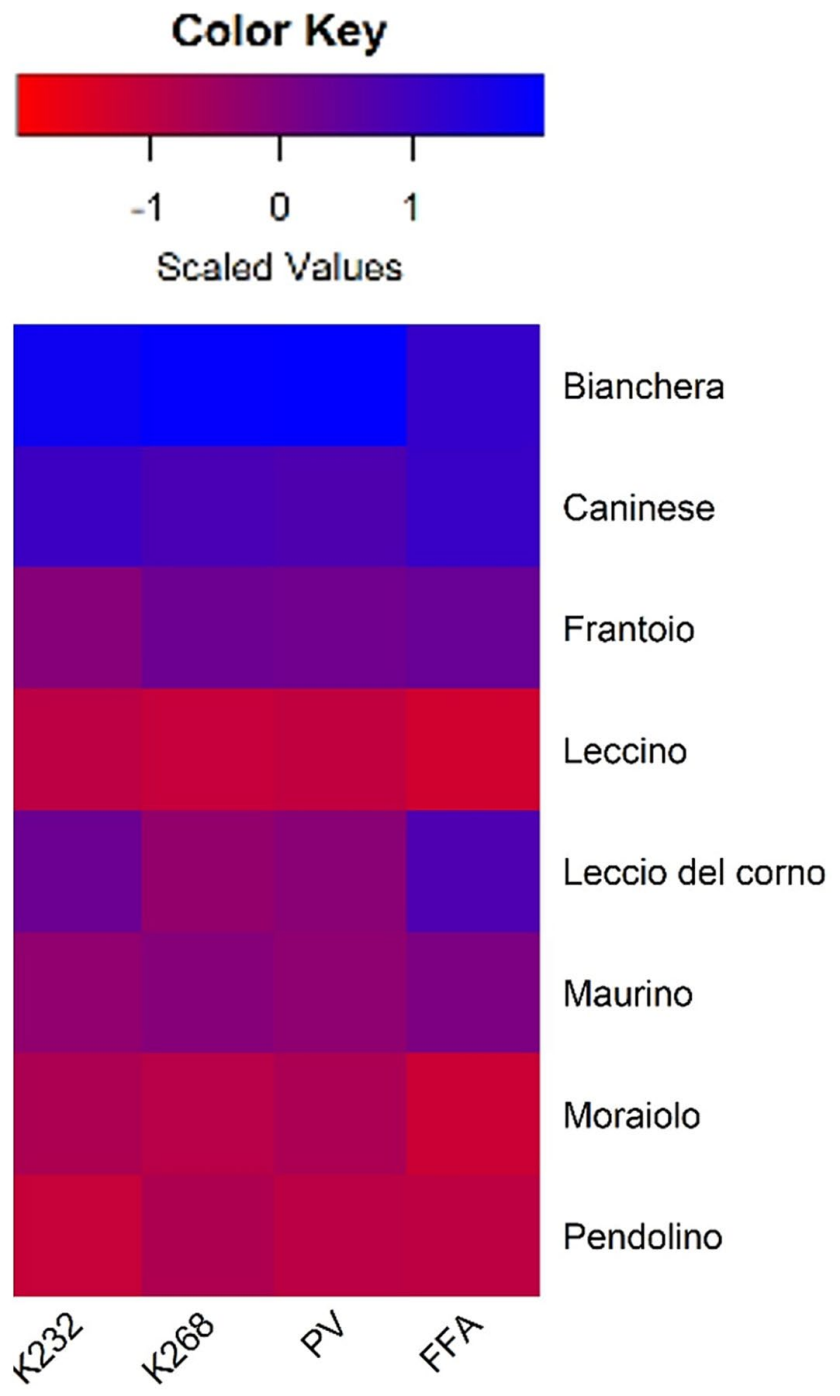
Heatmap representation of the fold change and scaled values [Log_2_(Stored/Control)] of the analyzed chemical parameters. Red indicates less than 0.5 fold differences in Stored compared to Control. Blue indicates higher than 1.5 fold in Stored compared to Control.

The heatmap representation of the fold change values (Figure 1) illustrates the impact of storage time on the analyzed chemical parameters. Intense blue hues indicate higher differences (more than 1.5-time fold) between Control and Stored, while deep red hues suggest lower differences (less than 0.5-time fold). Notably, cultivars Bianchera and Caninese exhibit the most pronounced disparities between Control and Stored, suggesting a higher sensitivity to storage time. In contrast, cultivars Leccino, Moraiolo, and Pendolino display milder differences in the analyzed parameters over the same period. The chemical parameter analysis underscores variations in oxidation rates among cultivars, with Leccino, Moraiolo, and Pendolino emerging as the most resistant, while Bianchera and Caninese demonstrate greater oxidative instability.

Our data suggests that storage induced significant differences in the changes of the oil chemical parameters depending on the olive cultivar. This is in line with the findings of Kıralan et al. (30), who reported differences in oxidation stability among olive oils from various cultivars in the East Mediterranean area of Turkey. The general increase in the evaluated parameters across all analyzed cultivars in the study indicates oxidation in the oil matrix during storage, consistent with the observations of Hbaieb et al. (31), who studied the effect of ripening and storage conditions of Chétoui and Arbequina olives.

### 3.2 Polar phenols compositions

A detailed examination of phenol composition in olive oil across different cultivars before and after the storage time (Table 2) provides some insights into the interplay between composition and stability. Comparing the phenol content in Control and Stored samples, there is a general decline in phenol content observed after the storage period, with some cultivars exhibiting a more pronounced decrease than others. Notably, Bianchera and Caninese, which were previously identified as potentially having low oxidative stability (as seen in Table 1), show a minimal decrease when comparing Control and Stored (52.75 vs. 49.4 and 47.87 vs. 44.60 mg/kg, respectively). Conversely, in Leccino and Pendolino, the reduction in the detected phenol amount is more pronounced than in Bianchera and Caninese (118.31 to 97.20, 131.45 to 113.34, and 117.85 to 87.76 mg/kg, respectively). The reduction effect observed in the current study aligns with findings from previous research. In a study by Criado et al. (32), a significant decrease in minor components, particularly within the phenolic fraction, was noted in commercial olive oil (Arbequina cultivar) after 12 months at room temperature.

**Table 2:** Polar phenol content (mg/kg) studied olive oil cultivars at zero (C) and after seven month of storage (S).

Cultivar	Time	Hydroxytyrosol	Tyrosol	Vanillic acid	Caffeic acid	Vanillin	p-coumaric acid	Ferulic acid	Rutin	Apigenin-7-glucoside	3,4-DHPEA-EDA	Pinoresinol	Cinnamic acid	Luteolin	Apigenin	Total
Bianchera	C	5.21 ± 0.34a	14.8 ± 4.79a	4.51 ± 0.81a	0.9 ± 0.11a	0.67 ± 0.13a	0.11 ± 0.01a	0.2 ± 0.03a	0.49 ± 0.01a	4.33 ± 0.2a	9.14 ± 0.83a	10.54 ± 1.57b	1.6 ± 0.14a	0.12 ± 0.07a	0.13 ± 0.02b	52.75 ± 5.23a
	S	5.64 ± 0.16a	16.64 ± 2.94b	4.75 ± 0.47a	0.77 ± 0.11a	0.68 ± 0.09a	0.1 ± 0.03a	0.12 ± 0.03b	0.37 ± 0.06b	1.97 ± 0.47b	2.22 ± 0.12b	14.57 ± 4.55a	0.81 ± 0.15b	0.15 ± 0.02a	0.61 ± 0.1a	49.4 ± 7.73b
Caninese	C	5.51 ± 1.42a	7.57 ± 1.8b	5.44 ± 0.21a	0.42 ± 0.08a	0.29 ± 0.04a	0.95 ± 0.16a	0.59 ± 0.11b	0.58 ± 0.07a	1.46 ± 0.07a	5.28 ± 0.82a	17.33 ± 3.93a	2.02 ± 0.37b	0.27 ± 0.05a	0.17 ± 0.02b	47.87 ± 7.46a
	S	6.63 ± 0.51b	9.56 ± 1.72a	3.72 ± 0.7b	0.38 ± 0.04a	0.12 ± 0.01b	0.92 ± 0.15a	0.72 ± 0.13a	0.53 ± 0.03a	0.31 ± 0.03b	2.93 ± 0.54b	12.65 ± 2.7b	6.46 ± 0.99a	0.25 ± 0.03a	0.42 ± 0.02a	44.60 ± 4.74b
Frantoio	C	25.13 ± 1.49b	0.5 ± 0.79b	3.01 ± 0.33b	0.09 ± 0.01a	0.42 ± 0.04a	0.11 ± 0.02b	1.03 ± 0.13b	0.22 ± 0.04a	4.34 ± 0.37a	14.28 ± 1.18a	8.11 ± 1.49a	3.35 ± 0.57a	0.64 ± 0.05a	0.86 ± 0.18a	62.06 ± 2.73a
	S	27.9 ± 0.92a	3.96 ± 0.19a	4.18 ± 1.12a	0.07 ± 0.01a	0.34 ± 0.07b	0.17 ± 0.01a	1.82 ± 0.35a	0.19 ± 0.03a	4.19 ± 0.35a	8.32 ± 2.2b	4.56 ± 0.47b	2.62 ± 0.56b	0.53 ± 0.04b	0.58 ± 0.14b	59.43 ± 6.34b
Leccino	C	38.72 ± 6.49b	5.24 ± 0.62a	8.47 ± 0.63a	6.1 ± 0.89a	3.19 ± 0.18a	3.75 ± 0.71a	3.9 ± 0.15a	0.05 ± 0.01a	2.87 ± 0.45b	27.18 ± 6.38b	7.46 ± 1.59b	5.94 ± 0.61a	1.99 ± 0.22a	3.44 ± 0.66a	118.31 ± 10.62a
	S	41.73 ± 1.34a	13.14 ± 0.39b	1.84 ± 0.37b	3.62 ± 0.69b	1.07 ± 0.24b	2.31 ± 0.22b	0.91 ± 0.11b	0.06 ± 0.03a	3.9 ± 0.35a	8.43 ± 2.16a	9.02 ± 1.35a	5.58 ± 0.98a	1.93 ± 0.29a	3.67 ± 0.48a	97.20 ± 6.34b
Leccio del corno	C	9.22 ± 0.92a	5.89 ± 0.94a	2.28 ± 0.08a	0.09 ± 0.02b	0.38 ± 0.06a	0.12 ± 0.02a	0.93 ± 0.16a	0.21 ± 0.06a	5.87 ± 0.46a	18.38 ± 1.14a	8.08 ± 1.27a	3.53 ± 0.3b	0.61 ± 0.06a	0.89 ± 0.17b	56.48 ± 8.77a
	S	5.75 ± 0.33b	4.58 ± 0.74b	1.32 ± 0.02b	0.17 ± 0.01a	0.33 ± 0.04b	0.11 ± 0.01a	0.84 ± 0.07a	0.21 ± 0.02a	4.17 ± 0.45b	16.64 ± 1.27b	4.3 ± 0.63b	8.3 ± 0.91a	0.58 ± 0.12a	1.16 ± 0.16a	48.46 ± 6.35b
Maurino	C	7.6 ± 0.81a	2.98 ± 0.36a	3.02 ± 0.27a	0.07 ± 0.02a	0.93 ± 0.12a	0.16 ± 0.03a	0.15 ± 0.03a	0.07 ± 0.02a	7.7 ± 1.12a	16.27 ± 2.87a	25.62 ± 2.02a	3.13 ± 0.45a	0.15 ± 0.01b	3.12 ± 0.23a	70.96 ± 4.84a
	S	4.44 ± 0.36b	1.95 ± 0.13b	1.01 ± 0.16b	0.04 ± 0.01b	0.31 ± 0.03b	0.13 ± 0.01b	0.03 ± 0.01b	0.07 ± 0.01a	5.91 ± 0.82b	13.72 ± 2.23b	6.97 ± 0.47b	2.09 ± 0.34b	0.56 ± 0.03a	0.39 ± 0.03b	37.61 ± 6.64b
Moraiolo	C	41.42 ± 10.82a	8.88 ± 0.55a	11.98 ± 1.02a	4.85 ± 0.28a	0.26 ± 0.05b	5.39 ± 0.97a	2.71 ± 0.45a	0.06 ± 0.04a	2.16 ± 0.35a	30.33 ± 6.28b	12.08 ± 0.25a	6.73 ± 0.5a	2.74 ± 0.49a	4.87 ± 0.41a	131.45 ± 16.63a
	S	53.56 ± 3.98b	15.14 ± 0.95b	6.21 ± 0.99b	2.88 ± 0.25b	0.47 ± 0.07a	2.1 ± 0.17b	1.42 ± 0.3b	0.08 ± 0.02a	0.65 ± 0.10b	9.52 ± 4.73a	6.54 ± 2.77b	6.36 ± 0.88a	3.53 ± 0.16a	4.88 ± 0.26a	113.34 ± 13.31b
Pendolino	C	39.79 ± 6.43a	6.1 ± 0.25a	9.64 ± 0.82a	5.44 ± 0.88a	0.43 ± 0.07a	3.99 ± 0.42a	4.48 ± 0.78a	0.42 ± 0.09a	2.69 ± 0.23a	23.15 ± 4.6b	11.07 ± 1.6a	5.74 ± 0.88a	1.82 ± 0.32a	3.08 ± 0.18a	117.85 ± 20.56a
	S	43.7 ± 2.53b	10.85 ± 0.14b	2.53 ± 0.6b	1.94 ± 0.22b	0.48 ± 0.06a	1.88 ± 0.5b	2.41 ± 0.39b	0.08 ± 0.01b	1.13 ± 0.23b	4.26 ± 1.74a	8.06 ± 0.96b	5.65 ± 0.73a	1.98 ± 0.16a	2.81 ± 0.35a	87.76 ± 9.34b

The results are presented as mean ± SD (*n* = 3). Different letters signify statistically significant differences (*p* < 0.05) between control and treated samples (Control and Stored). Total: Sum of all the quantified phenols.

In the samples characterized by higher oxidative stability, such as Leccino, Moraiolo, and Pendolino, a notable increase in hydroxytyrosol and tyrosol was observed (Annex 1), accompanied by a decrease in oleuropein hydrolyzed derivate (3,4-DHPEA-EDA). This trend parallels findings reported by Di Stefano and Melilli (33). The observed effects in the analyzed samples may stem from the degradation of 3,4-DHPEA-EDA, shedding light on the dynamic changes in phenolic composition during storage and their potential implications for oxidative stability.

Upon closer examination of the cultivars, samples related to higher oxidative stability had higher concentrations of hydroxytyrosol, tyrosol, vanillic acid, caffeic acid, p-coumaric acid, and ferulic acid. Notably, the antioxidant activity of 3,4-DHPEA-EDA, hydroxytyrosol, and phenyl acids (including caffeic acid, p-coumaric acid, ferulic acid, and vanillic acid) has been well-documented in the literature, emphasizing the potential contribution of these compounds to the observed oxidative stability in olive oil samples (34, 35).

These findings evidenced the impact of the cultivar on the phenolic content, which is crucial in the oxidative stability of the oil and its quality preservation along the storage time.

### 3.3 Multivariate analysis of the chemical quality parameters and polar phenol content

**Figure 2:**
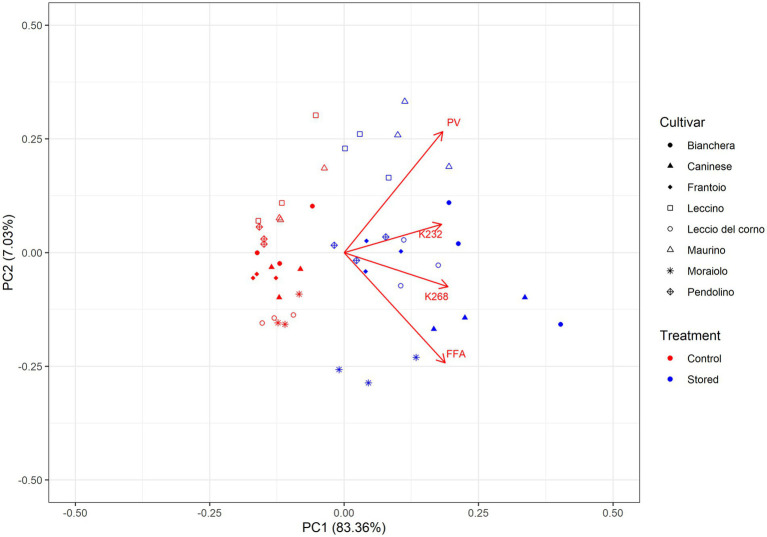
Principal component analysis (PCA) of the chemical quality parameters of the oil samples. The PCA consisted of the loading plot of PC1 versus PC2 and the score plot and distribution of the samples in the consensus space. In the score plot, each cultivar is represented by a different symbol. Symbols in red correspond to Control, while the blue color corresponds to Stored. Control: Oil analyzed at the beginning of the monitoring. Stored: Oil analyzed at the end of the trial.

Principal Component Analysis (PCA), as depicted in Figure 2, offered a comprehensive assessment of the intricate relationships between cultivars and the temporal effects of storage on their chemical quality parameters. At the same time, univariate analysis provided insights into cultivar oxidative stability. The multivariate analysis allowed for a nuanced understanding of how cultivar and storage differences influenced the overall composition of olive oil, revealing positive or negative correlations among the detected chemical quality parameters. The PCA accounted for a substantial portion, 90.38%, of the total variance, with variables positioned farther from the origin recognized for their significant impact on sample discrimination.

In the Principal Component Analysis (PCA), with PC1 explaining 83.35% and PC2 explaining 7.03% of the total variance, the distribution of samples is notable. The control samples are situated in the negative score plot region of PC1, indicating distinct separation from the stored samples, which are positioned to the right. The loadings, representing chemical quality parameters, exhibit a negative correlation with the control samples and a positive correlation with the stored samples. This suggests that the chemical composition associated with oxidative stability is different between control and stored samples. The control samples appear more aggregated in the negative region of PC1, indicating similarity, while the stored samples are more dispersed, reflecting greater variability. Furthermore, the highest score values for PC1 are attributed to samples considered less oxidative stable, emphasizing a potential association between the chemical composition and oxidative stability.

A general trend can be observed in Figure 2, where the evaluated chemical parameters (PV, FFA, K232, and K268) are positively correlated with the stored samples. This effect is in accordance with what is observed in Table 1, where an increase in these parameters is reported after the storage time. The data reveals a robust positive correlation between K232 and K268, and the stored samples were categorized as less oxidatively stable. This observation suggests that these specific values play a crucial role in distinguishing the oxidative stability among different cultivars, and the substantial decrease in phenolic compounds in these cultivars (Table 2) makes the oil more susceptible to oxidations, emphasizing the influence of storage conditions on the chemical composition of the samples. Studies performed by Ben-Hassine et al. (36) and Köseoğlu et al. (37) have reported analogous effects in stored samples, revealing a stronger association between chemical quality parameters and the stored samples. The evaluation by Köseoğlu et al. (37) showed that quality indices K232 and K268 values were mainly influenced by the storage. Meanwhile, the extraction system mainly influenced free acidity and peroxide value.

In previous studies, various olive varieties from the same geographical regions were effectively classified using models based on principal component analysis (PCA) (38). Additionally, it was observed that the phenolic fractions of the oil underwent quantitative changes based on cultivar (32). Consequently, the phenolic profile has been proposed as a tool for classifying olive oils according to cultivar (39, 40), although the establishment of a model suitable for cultivar identification and authenticity needs more extensive and prolonged studies (41).

**Figure 3:**
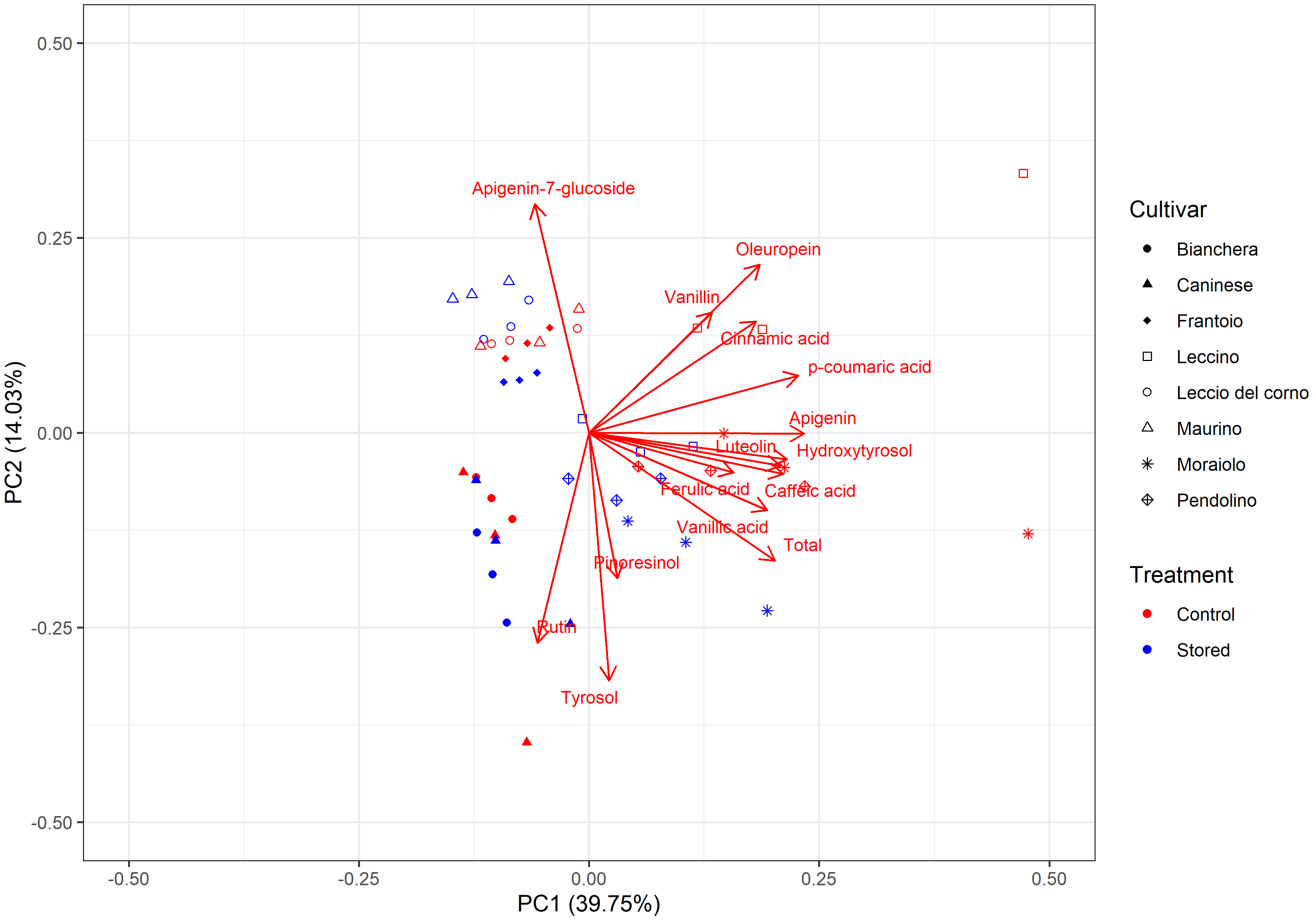
Principal component analysis (PCA) of the polar composition of various Italian olive oils. The PCA consisted of the loading plot of PC1 versus PC2 and the score plot and distribution of the samples in the consensus space. In the score plot, each cultivar is represented by a different symbol. Symbols in red correspond to Control, while the blue color corresponds to Stored. Control: Oil analyzed at the beginning of the monitoring. Stored: Oil analyzed at the end of the trial. Oleuropein: 3,4-DHPEA-EDA.

Principal component analysis (PCA), shown in Figure 3, was employed for a comprehensive evaluation of the interrelationships among cultivars and the temporal impact of storage on their phenol composition. While univariate analysis provided valuable insights into the oxidative stability of the cultivars, multivariate analysis enabled a more nuanced understanding of how cultivar and storage differences influenced the overall composition of olive oil and how the detected phenols are positively or negatively correlated with each other. The PCA explained a substantial portion, 53.78%, of the total variance. The variables positioned far from the origin are recognized to exert a significant impact on the discrimination of the samples.

PC1 accounted for 39.75% of the variability and showed a positive correlation of acid vanillic, p-coumaric, caffeic, and ferulic with hydroxytyrosol, luteolin, and apigenin. PC2 represented 14.03% of the total variance and showed a correlation between rutin, tyrosol, and pinoresinol but was negatively correlated with vanillin, 3,4-DHPEA-EDA, and apigenin-7-glucoside. This analysis indicates that tyrosol and 3,4-DHPEA-EDA are negatively correlated, as shown in Table 2. The cumulative content of assessed phenolic compounds exhibits a stronger correlation with samples characterized by high oxidative stability. This observation aligns with the findings presented in Table 2, where the reduction of phenols is less pronounced in Leccino, Moraiolo, and Pendolino varieties while the cumulative content of assessed phenolic compounds is negatively correlated with Bianchera and Caninese, stating that these cultivars have a higher phenolic reduction.

The individual scores for each principal component (PC) revealed the influence of the cultivar and storage on the phenol composition. In the individual scores, three distinct aggregations were discernible: the less oxidative stable group was Bianchera and Caninese, the intermediate oxidative stability group included Frantoio, Leccio del Corno, and Maurino, and the higher oxidative stability group was represented by Leccino, Moraiolo, and Pendolino. The positive PC1 scores associated with phenyl acids and secoiridoid derivatives are correlated with the samples hypothesized to be more oxidative stable. Within the positive scores in PC1, two distinct clusters can be observed: Control (red) and Stored (blue), indicating differences in storage time in Leccino, Moraiolo, and Pendolino. However, this cluster differentiation is not observed in the negative scores of the PC1, suggesting that the samples are very similar. Less oxidative stable samples showed positive PC2 scores, associated with a higher content of rutin, tyrosol, and pinoresinol, while the intermediate oxidative stability is characterized by a higher amount of apigenin-7-glucoside.

## 4 Conclusion

Chemical quality declined after storage, and storage time’s impact varied among cultivars. Bianchera and Caninese showed more marked differences in chemical quality between control and treated samples compared to the less affected Leccino, Moraiolo, and Pendolino. This suggests that the higher differences in the analyzed chemical parameters correlate with higher oxidation, emphasizing the importance of cultivar-specific conditions in storage. Phenol evaluation further supported these findings, indicating that cultivars with elevated levels of hydroxytyrosol, tyrosol, vanillic acid, caffeic acid, p-coumaric acid, and ferulic acid are associated with lower oxidation.

Multivariate analysis (PCA) revealed distinct correlations of phenols in the evaluated cultivars. The compounds rutin, tyrosol, and pinoresinol were positively correlated with Bianchera and Caninese, shown in the positive values of PC2. Alternatively, positive values in PC1 show the positive correlation of phenyl acids and secoiridoids derivates in the increased oxidative stability.

These findings not only underscore the significance of cultivar in maintaining olive oil’s oxidative stability but also emphasize that monitoring these particular phenols serves as an indicator of the oil’s overall quality and shelf life.

A practical suggestion involves recommending specific storage conditions tailored to different olive cultivars to achieve an extended shelf life. For example, colder temperatures may be beneficial for varieties like Bianchera and Caninese since a higher autoxidation rate is related to high temperature. The study’s limitations include the constrained control versus treated samples and storage duration. Additionally, proposing future research avenues, such as investigating the storage of diverse cultivars at different temperatures and monitoring phenolic and quality changes over time, can provide valuable insights for achieving a top quality one-year shelf life across all cultivars.

This research demonstrates how storage affects oil quality in relation to varietal distinctions. Nevertheless, it underscores the need for additional samples to validate these observations conclusively. Additionally, further research is needed since hydroxytyrosol and tyrosol are also released from oleuropein and ligstroside aglycones by esterases of vegetal and microbial origin. These findings indicate that oxidative stability is influenced not only by phenol composition and genotype but also by these enzymatic actions.

## Data availability statement

The raw data supporting the conclusions of this article will be made available by the authors, without undue reservation.

## Author contributions

MV: Data curation, Formal analysis, Software, Writing – original draft, Writing – review & editing. XL: Investigation, Methodology, Resources, Supervision, Visualization, Writing – review & editing. SB: Conceptualization, Supervision, Validation, Visualization, Writing – review & editing. PT: Conceptualization, Methodology, Project administration, Resources, Supervision, Validation, Visualization, Writing – review & editing. SW: Funding acquisition, Project administration, Resources, Supervision, Validation, Visualization, Writing – review & editing.
