# Supplementary figures and images for: Storage effect on olive oil phenols: cultivar-specific responses
Source: Front Nutr. 2024 Jul 10;11:1382551. doi: 10.3389/fnut.2024.1382551 (PMC11285335; doi:10.3389/fnut.2024.1382551)

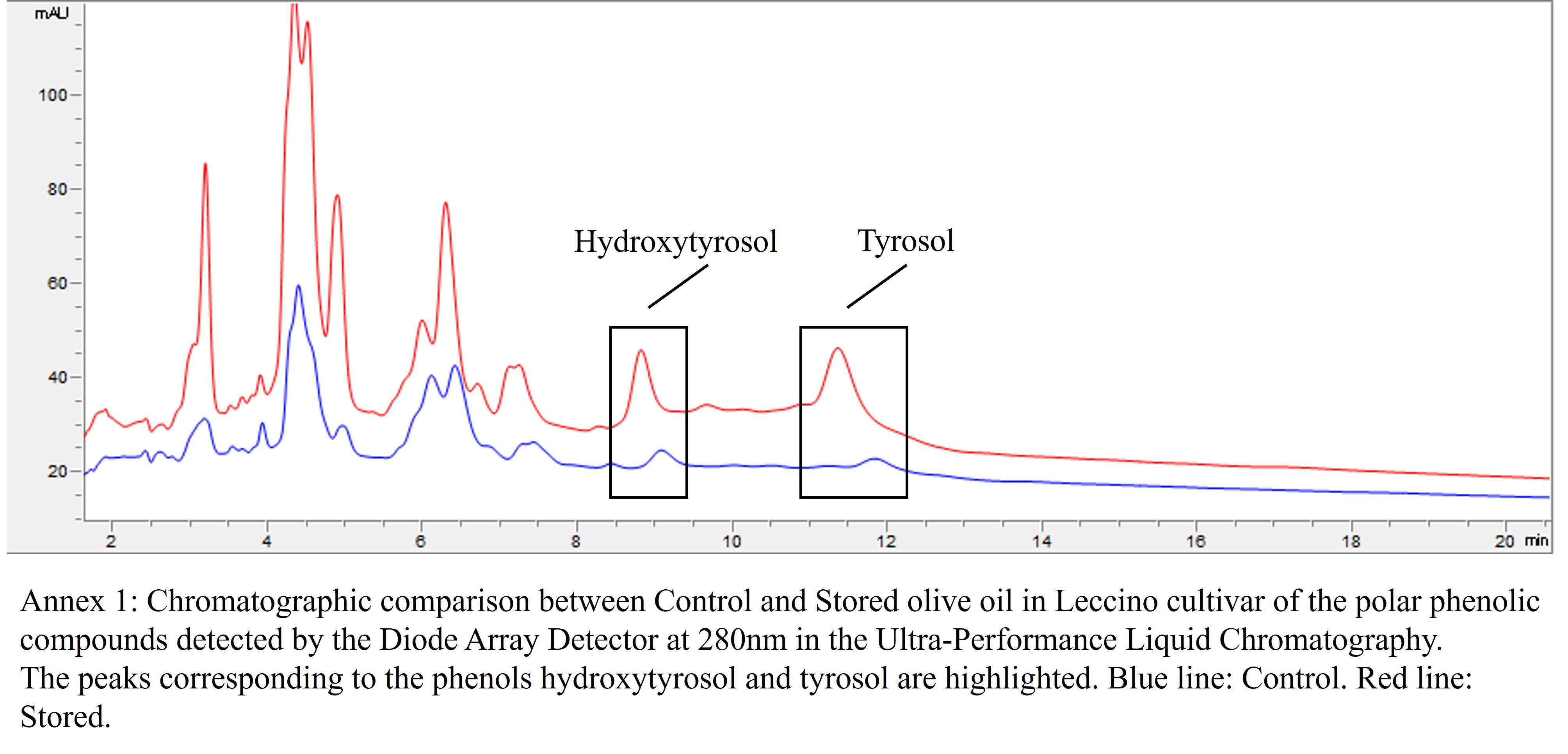

Supplement: Supplementary file 1 [file Image_1.JPEG]
